# Excessive Implant Wear Reaction Mimicking Malignant Tumors: A Rare Orthopedic Case Report

**DOI:** 10.3390/jcm14196949

**Published:** 2025-10-01

**Authors:** Lukas K. Kriechbaumer, Marian Mitterer, Patrick F. Marko, Sebastian Filipp, Christian Deininger, Eckhard Klieser, Andreas Hartmann, Thomas Freude

**Affiliations:** 1 University Clinic of Orthopaedics and Traumatology, Paracelsus Medical University, 5020 Salzburg, Austria; m.mitterer@salk.at (M.M.); p.marko@salk.at (P.F.M.); s.filipp@salk.at (S.F.); c.deininger@salk.at (C.D.); a.hartmann@salk.at (A.H.); t.freude@salk.at (T.F.); 2 Institute of Pathology, University Hospital Salzburg, Paracelsus Medical University, 5020 Salzburg, Austria; e.klieser@salk.at

**Keywords:** implant wear, adverse local tissue reaction (ALTR), metal-on-metal (MoM), metal-on-polyethylene (MoP), tumor-like lesion, periprosthetic joint infection (PJI), total hip arthroplasty (THA)

## Abstract

A 75-year-old patient was transferred to the oncology department due to the discovery of a large pelvic tumor compressing the femoral neurovascular bundle suspected to be of malignant origin. Further investigation revealed a rare complication related to a 27-year-old total hip arthroplasty (THA). The final diagnosis was a severe adverse local tissue reaction (ALTR) resulting from excessive implant wear—first from a metal-on-metal (MoM) bearing and later exacerbated by a revision to a metal-on-polyethylene (MoP) articulation. The clinical course was further complicated by periprosthetic joint infection (PJI). The patient underwent extensive tumor-like mass resection followed by two-stage revision arthroplasty. Despite these interventions, infection persisted, ultimately necessitating joint resection. This case highlights the rare but serious convergence of dreaded orthopedic complications (ALTR and PJI). It underscores the diagnostic challenge posed by wear-induced pseudotumors, which are rare even among arthroplasty specialists and are often unfamiliar to oncologists. This case illustrates the importance of early orthopedic evaluation, maintaining a high index of suspicion in atypical presentations, and invites further discussion about the interplay between ALTRs and infection risk in arthroplasty patients.

## 1. Introduction

Excessive wear reactions associated with orthopedic implants can present with clinical and radiological features that closely mimic malignant neoplasms. These lesions typically arise from adverse local tissue reactions (ALTRs) to wear debris—most commonly metal, but also polyethylene or ceramic particles—and are characterized by lymphocytic infiltration, soft tissue necrosis, and the formation of granulomatous lesions [1]. Although the exact pathophysiological mechanisms underlying these reactions remain incompletely understood, they pose significant diagnostic challenges, particularly in patients with long-standing implants. In such cases, wear-induced pseudotumors and extensive osteolysis can resemble malignancy or deep-seated infections, complicating clinical assessment and potentially delaying appropriate treatment. Early recognition and differentiation of these rare but serious complications are critical for guiding effective management strategies [2,3,4,5].

We report a rare and diagnostically challenging case involving a 75-year-old male patient initially suspected of having a pelvic malignancy based on clinical and radiologic assessment. Further investigation revealed an uncommon combination of complications related to a total hip arthroplasty (THA) performed 27 years earlier. The final diagnosis was an ALTR resulting from excessive implant wear—initially from a metal-on-metal (MoM) bearing surface and notably, later from a metal-on-polyethylene (MoP) articulation following revision surgery. This dual-pattern wear-induced ALTR was further complicated by a chronic periprosthetic joint infection (PJI), together closely mimicking a neoplastic process. This case highlights the diagnostic challenges posed by long-standing prosthetic implants.

The patient was informed that data concerning his case would be submitted for publication, and he consented.

## 2. Case History

**Figure 1:**
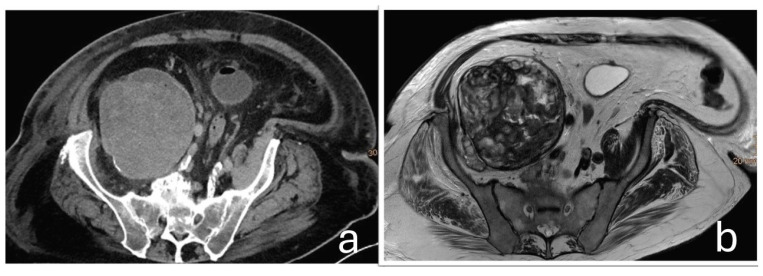
Axial CT (**a**) and T2-weighted MRI (**b**) images demonstrate a large, highly heterogeneous retroperitoneal mass in the right pelvis, measuring 15 × 13 × 11.5 cm, with both cystic and solid components and evidence of capsule formation. The lesion displaces adjacent anatomical structures and extends to the infrainguinal portion of the pectineus muscle.

A 75-year-old male patient was transferred to the oncology department of our hospital following the discovery of a large retroperitoneal mass on Computed Tomography (CT) and Magnetic Resonance Imaging (MRI). The lesion, measuring 15 × 13 × 11.5 cm with both cystic and solid components, was adherent to the iliac vessels and suspected to be malignant (Figure 1).

**Figure 2:**
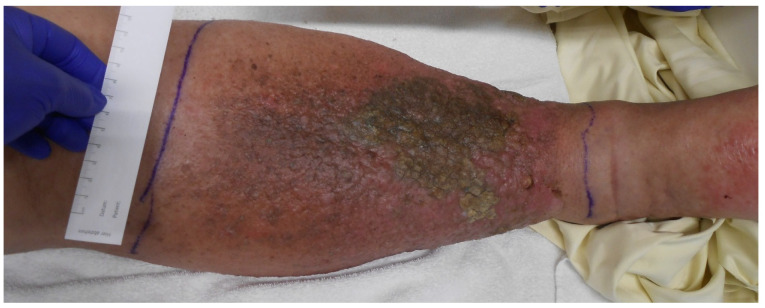
During the orthopedic consultation, venous edema and chronic lymphedema were observed. The erysipelas, initially present and treated with antibiotics, had largely resolved at the time of examination.

Imaging was initiated during an inpatient admission to another hospital in 2023 for markedly elevated inflammatory markers (C-reactive protein [CRP]: 32 mg/dL, leukocytes: 19 G/L) and erysipelas of the right lower extremity, for which the patient received antibiotic therapy (Cefuroxim and Clindamycin). Due to significant swelling of the right leg that developed over almost one year, further imaging (ultrasound, CT, and MRI) was performed, revealing an indeterminate pelvic mass on the right side, compressing the femoral neurovascular bundle at the level of the inguinal ligament. This resulted in venous congestion and elephantiasis of the right lower extremity (Figure 2).

The initial radiological impression suggested a hematoma. However, as additional specialties—including vascular surgery, angiology, and oncology—became involved, concerns grew regarding the possibility of a neoplastic process, particularly given the extent of the mass. Once this suspicion was raised, it proved difficult to definitively exclude a neoplastic etiology without histological confirmation. A full-body CT scan showed no evidence of additional malignancies.

The patient’s medical history is notable for a transient ischemic attack (TIA), obesity (body mass index: 37 kg/m^2^), arterial hypertension, type 2 diabetes mellitus, and hyperlipidemia (Fredrickson Type IIb). Additional comorbidities include aortic sclerosis, mild mitral and aortic valve insufficiency, hepatic steatosis, and benign prostatic hyperplasia. Collectively, these conditions represent a substantial cardiovascular and metabolic disease burden, consistent with an American Society of Anesthesiologists (ASA) physical status classification of III.

During the inpatient stay, the patient developed bilateral pulmonary embolism, necessitating temporary oxygen supplementation and observation in an intermediate care unit.

At the time of the orthopedic consultation at the University Clinic of Orthopaedics and Traumatology, Paracelsus Medical University of Salzburg, Austria, the patient’s cardiorespiratory condition had already significantly improved, and the inflammatory markers were regressing (C-reactive protein: 4 mg/dL; leukocytes: 7.4 G/L). Although the consultation was initially scheduled to obtain a biopsy of the tumor, attention was redirected to the patient’s long-standing hip arthroplasty.

In 1996, the patient underwent metal-on-metal (MoM) total hip arthroplasty (THA) for primary osteoarthritis at another hospital. A Bösch-type screw cup with a sandwich-type inlay—comprising a metal shell encased in a polyethylene liner—was implanted, along with a TiAl6V4 Proximal-Press-Fit (PPF) stem featuring a CCD angle of 131.5° (Biomet, Inc., Warsaw, IN, USA) and a 32 mm cobalt-chromium femoral head (XXL +12 mm). The acetabular component was positioned at an inclination angle of 46° and an anteversion of 18°. The postoperative course was uneventful, and the patient remained asymptomatic for 20 years following the procedure.

**Figure 3:**
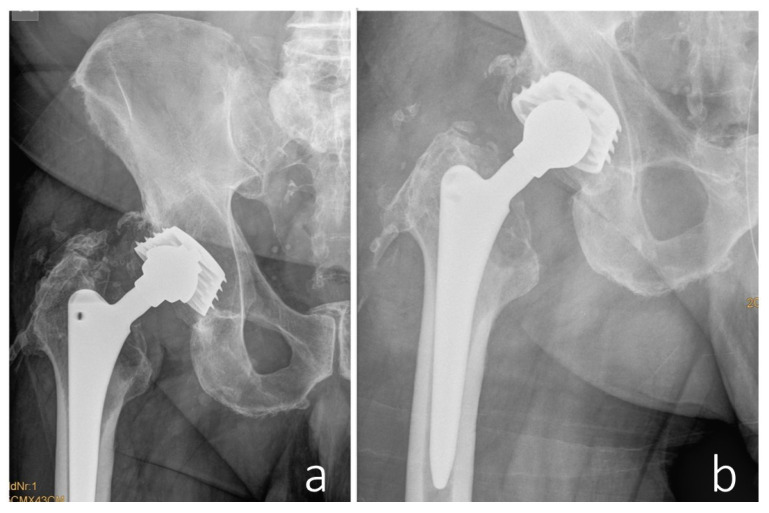
(**a**) The eccentric position of the prosthetic head is visible, indicating wear-related reaction of the sandwich-type inlay (metal shell encased in a polyethylene liner) in the main load-bearing zone. Additionally, heterotopic ossifications around the hip joint are observed. (**b**) The condition after the head/inlay exchange. The new metal head is now centrally positioned in the cross-linked polyethylene insert.

In 2017, a revision surgery was performed in the same hospital due to persistent hip joint pain and radiologically evident eccentric wear of the liner in the primary load-bearing zone. The liner was replaced with highly cross-linked polyethylene and a new metal head (Figure 3).

The local wear-induced reaction in the periarticular tissue was assessed as mild, and the bone-anchored prosthetic components were left in place. Prior to the revision hip surgery, serum metal ion analysis revealed elevated cobalt (8.7 µg/L) and chromium (1.22 µg/L) concentrations. Sonication, fluid and cyst wall cultures failed to grow bacteria. The patient’s postoperative recovery was again uneventful.

**Figure 4:**
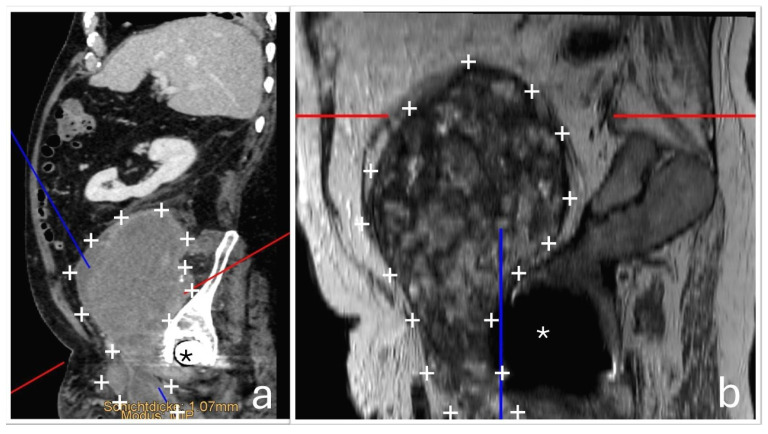
Additional reconstructed CT (**a**) and MRI (**b**) images demonstrate the close anatomical relationship between the pelvic mass (+) and the distally located total hip arthroplasty (*). The red and blue lines in the cross-sectional images indicate additional calculated planes that show the spatial proximity between the implant and the mass.

At the time of orthopedic examination, the hip joint was functional and non-painful. However, by reconstructing additional imaging planes from the existing CT and MRI datasets, the close anatomical relationship between the hip joint and the tumor mass could be visualized more clearly. (Figure 4).

A diagnostic joint aspiration was performed, revealing an elevated synovial leukocyte count (6000 cells/µL). The Synovasure^®^ test yielded inconclusive results, likely influenced by the presence of hematoma; additionally, metal debris associated with metallosis can interfere with test accuracy and lead to false-negative outcomes. No bacterial growth was detected in synovial fluid cultures, while the patient was still receiving antibiotic therapy, potentially limiting the sensitivity of the test. Cobalt and chromium blood levels were not obtained, as the presence of an adverse local tissue reaction was evident, and the results of such testing would not have influenced the therapeutic decision-making or altered the planned management.

**Figure 5:**
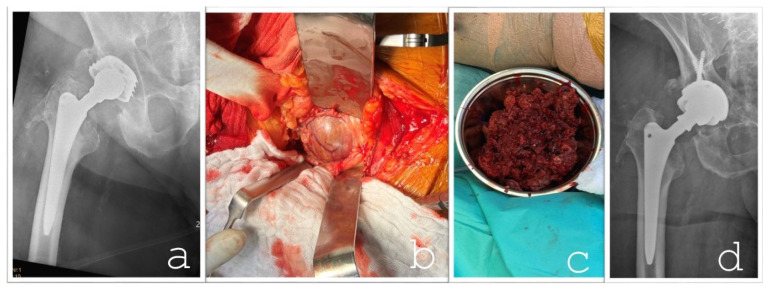
(**a**) The preoperative bony condition, highlighting mild bone loss along the proximal femur (specifically at the lesser trochanter in Gruen zone 7). The femoral stem remains well-fixed, particularly in the diaphyseal region. Additionally, a cystic lesion is observed at the base of the acetabular cup, which was more pronounced on the CT scan. (**b**) The capsule of a large mass in the pelvic cavity, exposed through the pararectal approach. The capsule contains a mixed composition, predominantly loose synovial tissue and organized hematoma (**c**). (**d**) The radiographic findings following the femoral head, inlay, and acetabular component revision.

The differential diagnosis pointed towards an excessive wear reaction from the hip implant, which had been in place for over 27 years. The reaction was thought to be the source of the tumor-like mass. The patient underwent surgery via two approaches: a pararectus approach to excise the main tumor, leaving a small residual mass adherent to the inguinal vessels, and an anterolateral approach for a revision of the hip joint. The procedures were performed by orthopedic and trauma surgeons, with vascular surgeons available on standby if required. An intraoperative histological frozen section revealed no indication of the presence of a malignant tumor. Macroscopic examination revealed only mild trunnion wear and corrosion at the femoral head–neck junction. Black pitting was observed at the head–neck junction; however, no other obvious surface damage was noted on the trunnion or on the corresponding contact area of the femoral head. As the femoral stem remained well-fixed intraoperatively, it was retained. The femoral head was replaced with a new ceramic component (Bioball^®^, Merete Medical GmbH, Berlin, Germany). Due to the unavailability of the original inlay and the presence of lytic cysts in the acetabulum, the acetabular component was revised. A new acetabular cup (Lima Delta-One TT; Lima Corporate, Villanova di San Daniele del Friuli, Italy) was implanted, secured with three bone screws and an angled spacer, along with an elevated rim liner. To optimize wear resistance and longevity, a highly cross-linked polyethylene liner infused with vitamin E was employed (Figure 5). Multiple tissue samples were taken during surgery, confirming the presence of an implant wear reaction.

**Figure 6:**
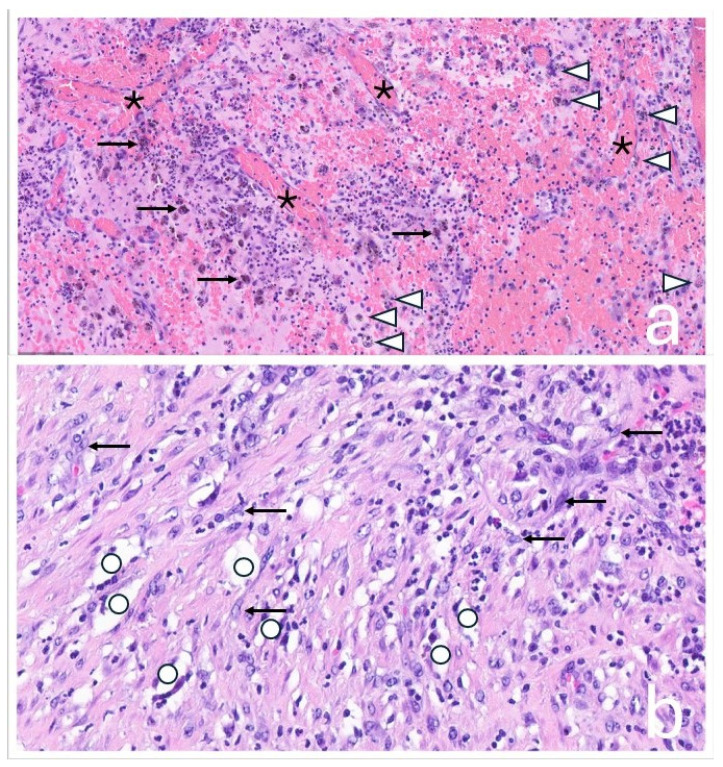
(**a**) Hematoxylin and eosin (H&E) staining at 200× magnification. It demonstrates a mixed inflammatory response. Numerous neutrophilic granulocytes and areas of erythrocyte extravasation are visible. Macrophages are prominent, including hemosiderin-laden macrophages (identified by brown pigment, →) and macrophages containing metal debris (black pigment, ∆). Additionally, there is extensive capillary proliferation (*), indicative of a chronic inflammatory process. (**b**) H&E staining at 400× magnification. It reveals a granulomatous inflammatory reaction with increased numbers of macrophages, predominantly with an epithelioid phenotype (←), and so-called ‘ghost particles’ (○): Polyethylene appears optically empty in H&E staining and presents as narrow, spindle-shaped or roundish voids, often without coloration (as polyethylene is not stainable).

The tissue was macroscopically trimmed and representatively embedded in paraffin. Sections were stained with hematoxylin and eosin and examined by light microscopy. Histological analysis showed a mixed chronic and active inflammatory response with metal and polyethylene wear particles, necrosis, and hemorrhage. The surrounding fibrotic capsule exhibited a lymphohistiocytic and florid inflammation. Findings were consistent with an organizing, encapsulated hematoma, without evidence of neoplastic infiltrates (Figure 6).

**Figure 7:**
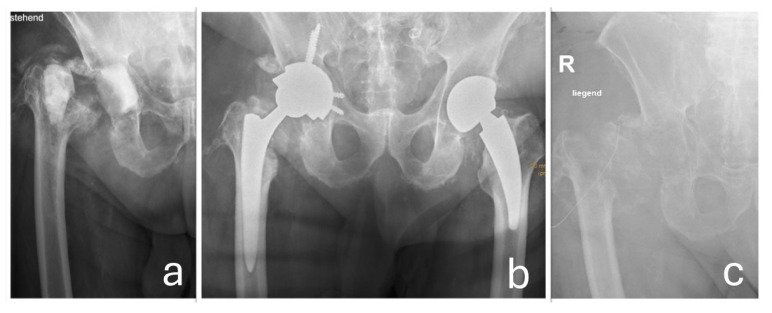
(**a**) The status post explantation of the prosthetic components with a non-articulating cement spacer in place. (**b**) The situation following delayed reimplantation of the total hip arthroplasty. Due to recurrent infection, a second explantation of the hip prosthesis was required, as shown in (**c**).

Postoperatively, there was notable improvement in lower extremity swelling, attributed to decompression of the neurovascular bundle. However, further diagnostic workup revealed a bacterial infection caused by methicillin-resistant *Staphylococcus aureus* (MRSA), confirmed through implant sonication, tissue cultures, and PCR analysis. Targeted antibiotic therapy was initiated according to the antibiogram, but the infection persisted despite appropriate treatment. As a result, a two-stage revision procedure was performed. This included thorough resection of residual pseudotumor tissue surrounding the neurovascular bundle and placement of a temporary, non-articulating, antibiotic-loaded cement spacer. Reimplantation was postponed for six months due to additional complications, including a nosocomial pulmonary infection and urinary tract infection caused by *E. coli* and *Pseudomonas*. Once these infections were resolved, reimplantation was successfully performed using a Lima Delta Multihole TT acetabular component, secured with bone screws, an angled spacer, a protrusively designed liner, a 36 mm XL ceramic femoral head, and an SL-Plus MIA high-offset femoral stem (Smith & Nephew). Unfortunately, reinfection with *Enterobacter cloacae* complex occurred post-reimplantation, ultimately necessitating joint resection (Figure 7).

## 3. Discussion

Excessive implant wear reactions are uncommon but well-documented, particularly in long-term total hip arthroplasty (THA). Pseudotumor formation is most frequently associated with MoM bearing surfaces or results from mechanically assisted crevice corrosion (MACC), which typically occurs at modular junctions—such as the head-neck or neck-stem taper—especially when cobalt-chromium components are involved. ALTRs remain a principal factor in the declining use of MoM implants [6,7,8,9].

These reactions can result in the formation of masses and granulomas, which may be radiologically indistinguishable from malignancies, making clinical diagnosis difficult. A connection track between the joint space and the pseudotumor may also collapse and therefore make the diagnosis more challenging [10].

If such pseudotumors become large enough, they can compress the surrounding tissue and cause multiple complications (swelling, palsy, necrosis, constipation, pain, …) [11]. In the present case, compression of the femoral neurovascular bundle was among the key clinical findings. A substantial pelvic mass was initially presumed to be a hematoma but was ultimately misdiagnosed as a malignant tumor, prompting an oncology referral.

Given the patient’s 27-year history of total hip arthroplasty, the absence of systemic signs of malignancy should have raised suspicion for an implant-related complication. However, the previous revision surgery six years earlier—during which implant wear was only moderate—may have diverted clinical attention away from this possibility. These types of cases are rare—even for orthopedic surgeons specializing in arthroplasty—and the radiological findings may be misinterpreted not only by oncologists, but also by radiologists and even orthopedic surgeons, given the unusual nature of the presentation. Early orthopedic consultation is essential to avoid misdiagnosis and unnecessary treatment, particularly when evaluating atypical pelvic or periprosthetic masses in patients with long-standing implants.

The diagnostic process was further complicated by an MRSA-associated PJI, which was not initially detected through blood cultures or joint aspiration. Such false-negative results are not uncommon in cases of low-grade, chronic infections associated with implant wear particles and ongoing antibiotic therapy. Although the exact source of the PJI remains unclear, hematogenous seeding during an episode of bacteremia—most likely originating from the erysipelas—is considered the most probable route of infection. Additionally, ALTRs can modulate the local immune response, potentially impairing the body’s ability to combat infections. Studies have shown that persistent infections can suppress immune cell activation, leading to decreased T-cell proliferation and tissue infiltration, which may contribute to the development and persistence of PJI [12,13]. Although these immunomodulatory effects cannot be directly assessed in the present case, they may have contributed to the patient’s apparent immunological vulnerability. While the patient exhibited systemic risk factors for infection—such as obesity and type 2 diabetes mellitus—the occurrence of multiple distinct infections, including erysipelas, PJI, pulmonary infection, and urinary tract infections, caused by different pathogens (MRSA, *E. coli*, *Pseudomonas*, and *Enterobacter cloacae* complex), is striking and raises concern for a potential underlying immune system impairment.

Despite undergoing a two-stage revision procedure, PJI reoccurred, ultimately necessitating joint resection.

## 4. Summary

This rare case highlights the complex interplay of multiple serious complications following total hip arthroplasty, including implant wear reactions mimicking malignancy, ALTRs from both MoM and MoP bearings, and PJI. The case underscores the diagnostic challenge posed by wear-induced masses, particularly in patients with long-standing implants, where such presentations can closely resemble malignant tumors. These manifestations are uncommon, even among arthroplasty specialists, and are virtually unknown to most oncologists, increasing the risk of misdiagnosis and inappropriate treatment.

Wear particles from MoP implants can provoke inflammatory responses similar to those seen with MoM articulations. The resulting local inflammation, immune modulation, tissue destruction, and potential prosthetic instability may create a biologic environment conducive to PJI. In this case, both an extensive implant wear reaction and an underlying infection were present. Notably, joint aspiration did not initially reveal the infection, and despite a two-stage revision procedure, the patient ultimately required joint resection. The large exposed wound surface involving the pelvis and hip joint may have contributed to the poor outcome.

This case emphasizes the importance of early orthopedic evaluation, a high index of suspicion in atypical cases, and a multidisciplinary approach to avoid diagnostic pitfalls. A better understanding of ALTRs and their contribution to PJI may help improve outcomes and guide future management strategies.

## Data Availability

The original contributions presented in this study are included in the article. Further inquiries can be directed to the corresponding author.
